# Investigating the Mechanism of Edible Medicinal Plants Against Squamous Cell Carcinomas Based on Network Pharmacology, Bioinformatics, and Molecular Dynamics Simulation

**DOI:** 10.3390/ijms27052141

**Published:** 2026-02-25

**Authors:** Shanfeng Liang, Shunzhen Yu, Xudong Tang

**Affiliations:** 1 School of Pharmacy, Gansu University of Chinese Medicine, Lanzhou 730000, China; 15865290230@163.com (S.L.); 18297717183@163.com (S.Y.); 2 Shenzhen Research Institute of Lanzhou University, Shenzhen 518107, China

**Keywords:** edible medicinal plants, squamous cancer, network pharmacology, bioinformatics, matrix metalloproteinase-1

## Abstract

This study utilized network pharmacology, bioinformatics, along with machine learning to investigate the multi-target synergistic anti-cancer mechanisms of three edible medicinal plants (EMPs)—mulberry leaf, lotus leaf, and sea buckthorn—against oral and esophageal squamous cell carcinomas (OSCC and ESCC). We identified potential active constituents and their targets through mining Traditional Chinese Medicine Systems Pharmacology (TCMSP) and Swiss Target Prediction databases. Concurrently, integration with differential expression profiles and co-expression modules identified crucial intersection targets between the EMPs and these two cancers. Subsequent machine learning algorithms and cross-cancer analysis consistently identified Matrix Metalloproteinase-1 (*MMP1*) as a critical hub gene. Its overexpression is closely associated with tumor invasion and metastasis. Molecular simulations revealed stable binding interactions between active constituents from three EMPs and hub proteins. Furthermore, research on immune cell infiltration suggested that the active components of three EMPs may impact the tumor immune microenvironment in both OSCC and ESCC through the regulation of pivotal gene expression. Collectively, this work systematically elucidates the molecular basis underlying the multi-target, multi-pathway synergistic anti-cancer effects of these EMPs, providing a theoretical foundation for developing natural drugs against these squamous cell carcinomas.

## 1. Introduction

Squamous cell carcinoma (SCC), an extremely aggressive malignancy arising from squamous epithelium, comprises over 90% of oral and esophageal cancer cases [1,2,3]. Although surgery combined with radiotherapy can provide partial relief, toxic side effects, high recurrence rate, and economic burden remain clinical pain points [4]. Therefore, exploring the development of novel drug candidates with reliable efficacy, low toxicity and side effects, as well as good economic benefits, is an urgent need in current society.

Being an essential element of China’s traditional medical system, edible medicinal plants (EMPs) possess both medicinal and edible properties, offering unique potential in tumor prevention and treatment due to their low toxicity, high safety, and multi-target regulatory properties [5]. Existing studies have confirmed that the three EMPs, namely mulberry leaf, lotus leaf, and sea buckthorn, explored in this study, have significant antitumor effects. Among them, mulberry leaf extract exhibits inhibitory activity against hepatocellular carcinoma cell proliferation [6,7]. Concurrently, research also indicates that mulberry leaf lectin promotes apoptosis in cancerous cells [8]. Flavonoids derived from lotus leaves suppress lung cancer progression by targeting the ROS/p38 MAPK pathway [9]. By targeting TGF-β1-induced Erk1/2 and SMAD3, lotus leaf alcohol extract hinders breast cancer cell migration [10]. Sea buckthorn proanthocyanidins can target fatty acid synthase to induce apoptosis [11]. And HRWP-A, derived from sea buckthorn berries, can effectively control the growth of lung cancer cells [12]. However, previous investigations have largely been limited to studying singular components or particular cancer species, and the common targets, immunoregulatory mechanisms, and cross-cancer synergistic effects of three EMPs against squamous carcinoma have not been systematically analyzed.

As a new paradigm of systemic medicine research, network pharmacology has realized a paradigm shift from the linear model of “single drug–single target” to the systemic research model involving “diverse compounds, targets, and pathways”. This paradigm shift is not only highly compatible with a “whole view” in traditional Chinese medicine (TCM) but also provides methodological support for analyzing the multidimensional network of “components-targets-pathways” in the synergistic effect of TCM [13,14]. It is worth noting that the breakthroughs in bioinformatics technology, especially the deep integration of machine learning algorithms and molecular docking technology, have significantly enhanced the prediction accuracy and analysis depth of drug-target interaction networks [15]. This multidisciplinary research approach enhances our understanding of the synergistic effects involved in complex drug systems, characterized by multiple components, pathways, and targets.

**Figure 1:**
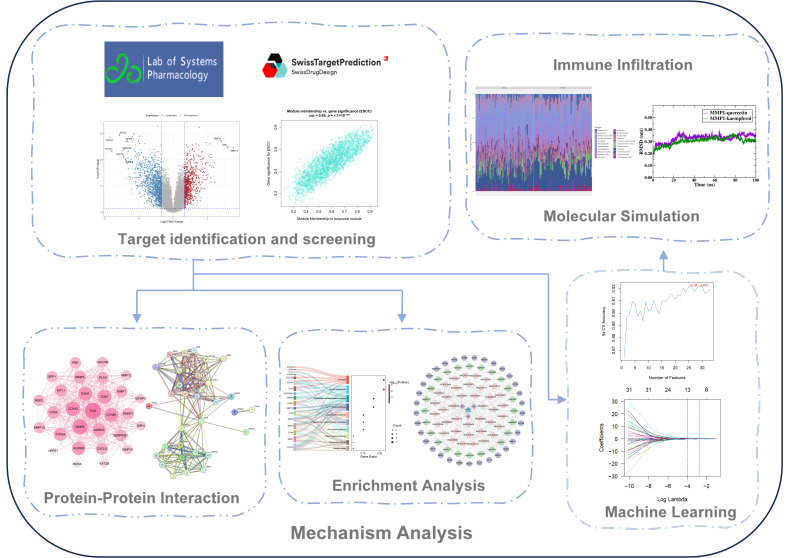
Experimental design.

Currently, there are not many studies exploring how mulberry leaves, lotus leaves, and sea buckthorn work in treating SCC. Our research employed network pharmacology along with bioinformatics to analyze public OSCC and ESCC datasets, exploring the key ingredients and workings of three EMPs against squamous carcinoma. The research elucidated the pharmacological foundation of the three EMPs and their workings, laying the theoretical foundation for their subsequent experimental validation and clinical translational research. Figure 1 illustrates a schematic diagram of our workflow.

## 2. Results

### 2.1. Key Constituents and Targets Within EMPs

We determined 66 key components, as well as 788 targets from three EMPs using TCMSP and Swiss Target Prediction databases (Supplementary Table S1).

### 2.2. OSCC Therapeutic Targets

**Figure 2:**
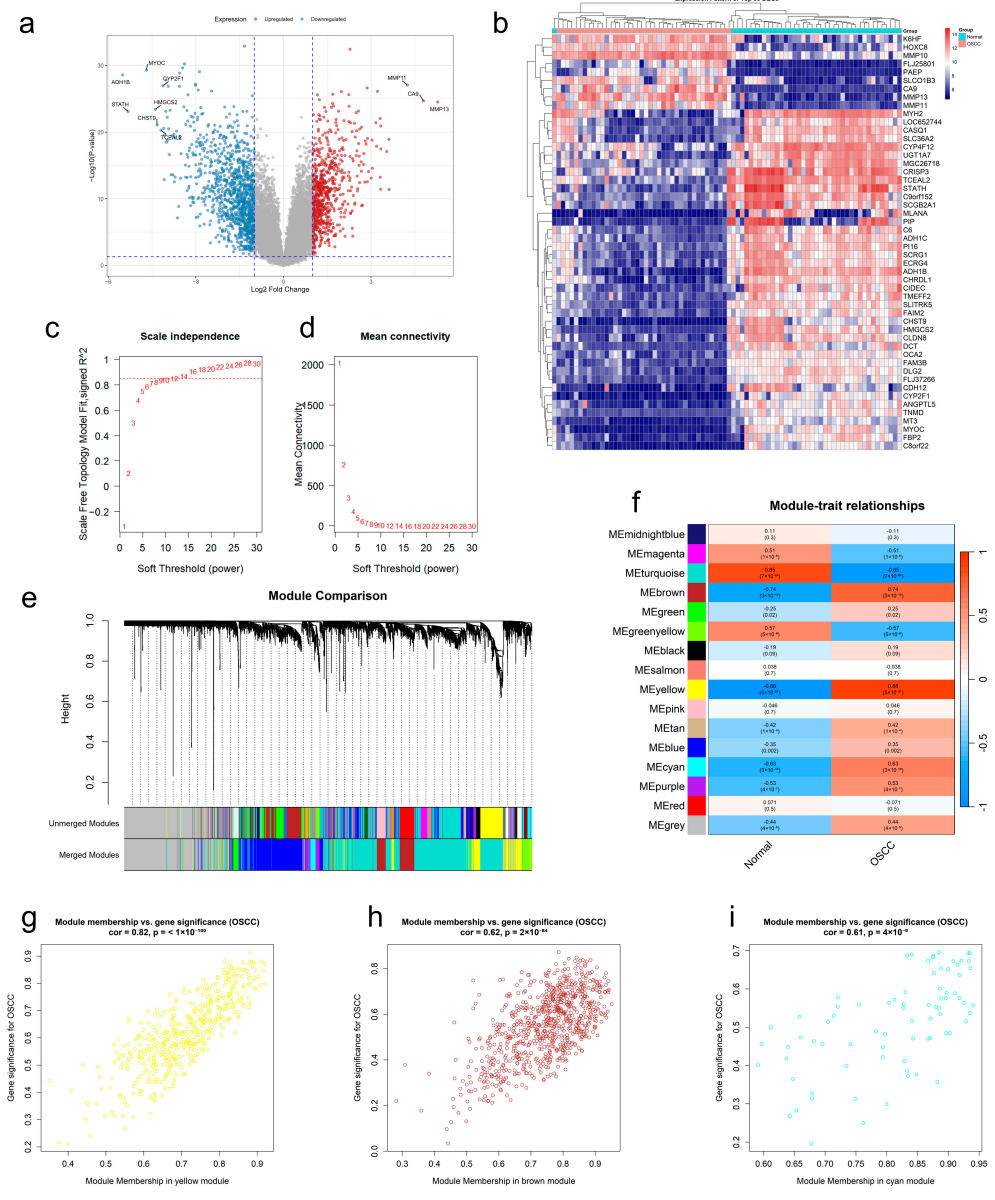
Acquisition of OSCC targets. (**a**) Volcanic map showing DEGs. (**b**) Top 50 DEGs heatmap. (**c**,**d**) Scale independence and average connectivity analysis in WGCNA. (**e**) WGCNA modules identified by color. (**f**) Module–trait association analysis plot for 16 modules. (**g**–**i**) Scatterplot showing gene significance vs. module membership about key modules (yellow, brown, and cyan).

**Figure 3:**
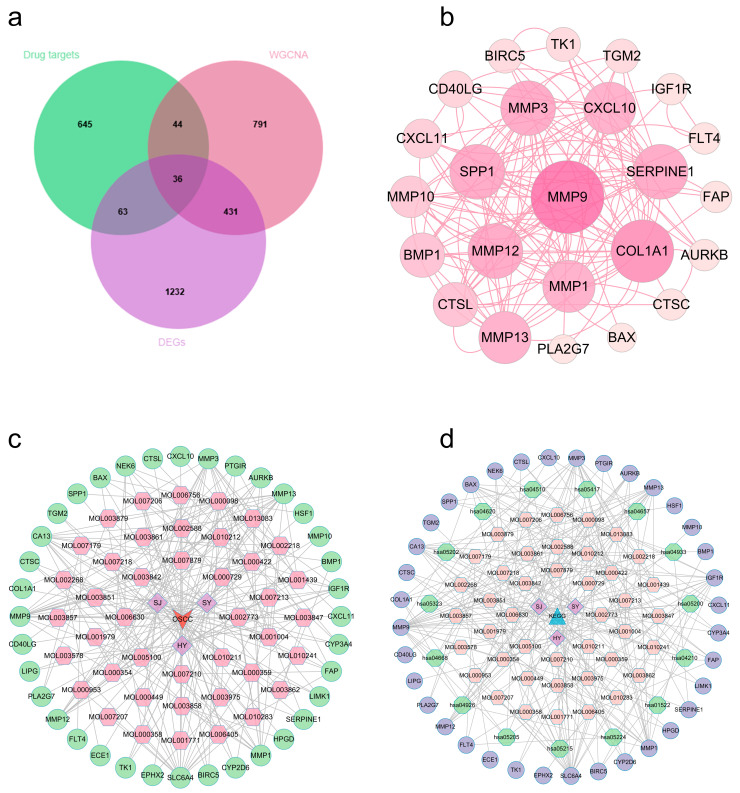
PPI and network visualization (OSCC). (**a**) Target predicted Venn diagram. (**b**) Raw protein–protein interaction networks for targets. (**c**) Drug–disease network: arrows represent oral squamous carcinoma, quadrilaterals represent the three EMPs (HY: lotus leaf, SY: mulberry leaf, SJ: sea buckthorn), hexagons represent the active ingredients, and circles represent the common targets. (**d**) Ingredient-target-pathway network: triangles represent KEGG, quadrilaterals represent EMPs (HY: lotus leaf, SY: mulberry leaf, SJ: sea buckthorn), octagons represent active ingredients, hexagons represent signaling pathways, and circles represent common targets.

After normalized preprocessing on the GSE37991 dataset, we found 1762 differentially expressed genes (DEGs) through a differential expression study, then displayed them by volcano and heat figures (Figure 2a,b). Subsequently, we employed Weighted Gene Co-Expression Network Analysis (WGCNA) to identify crucial modules linked to OSCC. We found that the best soft threshold for our analysis was 12, guided by the principles of scale independence and average connectivity (Figure 2c,d). After creating the co-expression network, we identified modules by the dynamic tree-cutting method. In the end, we combined similar modules to obtain 16 independent modules (Figure 2e). Module–trait association analysis showed that the yellow, brown, and cyan modules had the most significant and stable correlations with OSCC versus non-OSCC phenotypes (Figure 2f). Further analysis confirmed that gene significance (GS) for those genes in our above modules was highly and positively correlated with module membership (MM) (Figure 2g–i). The above studies indicated that there are 1302 potential regulatory targets of OSCC. Ultimately, 36 potential therapeutic targets (Supplementary Table S2) were screened by integrating OSCC differentially expressed genes, key module genes, and active component targets of EMPs (Figure 3a).

### 2.3. Protein–Protein Interaction (PPI) Network and Multidimensional Regulatory Network Construction (OSCC)

We added the identified intersecting targets to STRING platform for building one PPI graph. Cytoscape 3.10.3 was employed for visualizing it (Figure 3b). In this graph, nodes representing target proteins with higher degree values are visualized larger, indicating their more critical role in the treatment of OSCC using the three EMPs. Furthermore, we built and visualized a network that connects drugs, components, diseases, and targets (Figure 3c). Several components (e.g., quercetin, kaempferol, isorhamnetin, norartocarpetin, and inophyllum E), which have the top degree values, may play critical roles in the treatment of OSCC through multi-target synergism (Supplementary Table S4).

### 2.4. OSCC Target Functional Annotation

**Figure 4:**
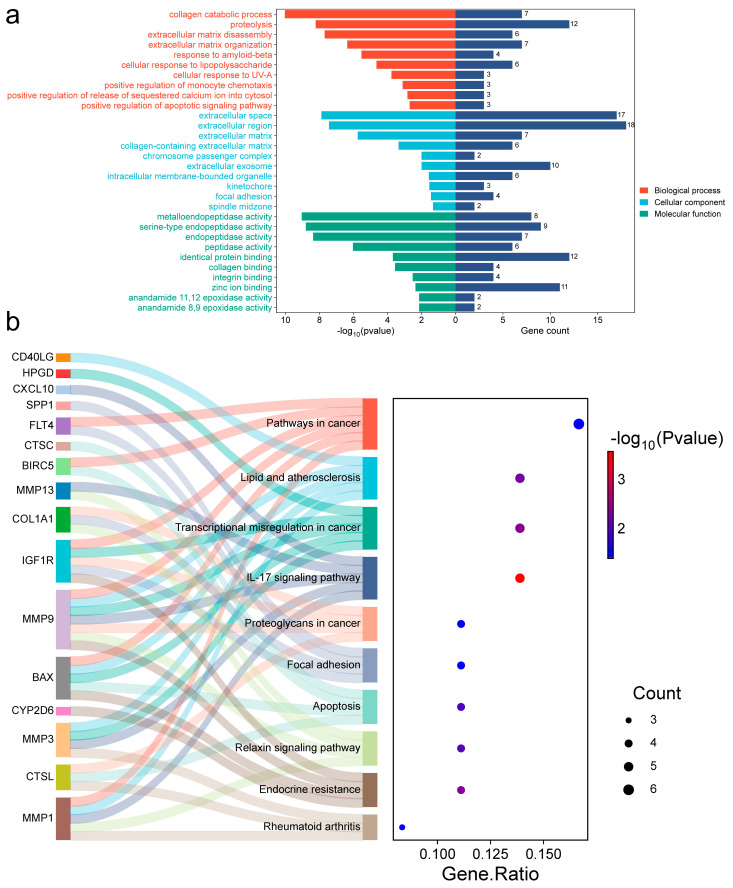
GO and KEGG analysis (OSCC). (**a**) GO profiling bilateral bar graph. (**b**) KEGG profiling bubble graph.

To investigate the underlying mechanism, we further analyzed those 36 common targets. Gene Ontology (GO) analysis (Figure 4a) revealed biological processes (BP) mostly focused on collagen catabolism, proteolysis, and extracellular matrix disassembly. Cellular components (CC) showed significant enrichment in the extracellular matrix, region, and space, among others. Molecular function (MF) involved metalloendopeptidase, serine-type endopeptidase, and endopeptidase activity, among others. Kyoto Encyclopedia of Genes and Genomes (KEGG) analysis (Figure 4b) showed that these targets were significantly enriched in the transcriptional misregulation in cancer, endocrine resistance, IL-17 signaling pathway, and other pathways (Supplementary Table S6). A detailed mapping of candidate hub genes to their associated enriched KEGG pathways is provided in Supplementary Table S7, clarifying their potential functional roles. Using Cytoscape 3.10.3, we built constituent-target-pathway networks of the three EMPs for OSCC treatment based on these results (Figure 3d).

### 2.5. Machine Learning Screening of Pivotal Genes (OSCC)

**Figure 5:**
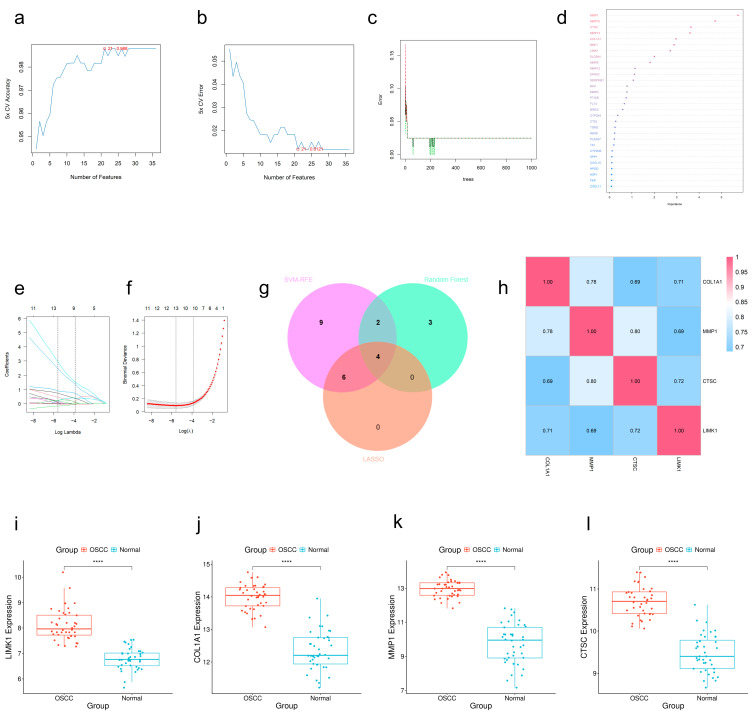
Machine learning-based hub gene screening. (**a**,**b**) 5-fold cross-validation accuracy vs. error rate curves for the SVM-RFE algorithm. (**c**,**d**) Error rate curves vs. significance assessment for the random forest algorithm. (**e**,**f**) Coefficients vs. regularized path plots for LASSO regression. (**g**) Venn diagram for key hub gene screening. (**h**) Heatmap analysis of expression correlation among hub genes. (**i**–**l**) Box plots of hub gene expression levels based on the GSE37991 dataset (****: *p* < 0.0001).

To rigorously identify hub genes, we applied three machine learning algorithms to the 36 candidate targets using the GSE37991 dataset. A nested 5-fold cross-validation framework with stratification was implemented to prevent overfitting and evaluate generalizability. We screened twenty-one highly distinguishable genes, *LIMK1*, *CTSC*, *MMP1*, *CYP2D6*, *COL1A1*, *BIRC5*, *BMP1*, *CXCL11*, *MMP3*, *SPP1*, *CD40LG*, *CTSL*, *AURKB*, *FAP*, *NEK6*, *PTGIR*, *SLC6A4*, *FLT4*, *HPGD*, *TGM2*, and *MMP9* using the support vector machine-recursive feature elimination (SVM-RFE) algorithm (Figure 5a,b). Additionally, the random forest (RF) algorithm revealed the top nine genes ranked by significant scores (*MMP1*, *MMP10*, *CTSC*, *COL1A1*, *BMP1*, *LIMK1*, *MMP13*, *EPHX2*, *MMP9*) (Figure 5c,d). LASSO analysis determined ten core target genes (*BIRC5*, *COL1A1*, *CTSC*, *CYP2D6*, *LIMK1*, *NEK6*, *CD40LG*, *FAP*, *MMP1*, *SPP1*) (Figure 5e,f). The intersection results of the three methods showed that *COL1A1*, *MMP1*, *CTSC*, and *LIMK1* played core roles in OSCC treatment (Figure 5g). Gene correlation and expression analysis demonstrated (Figure 5h–l) that these genes showed strong correlation with each other and significant expression differences between the disease and normal groups. SHAP (Shapley Additive exPlanations) analysis was performed on the final RF model, confirming the stability and interpretability of these feature selections (Figure S4).

### 2.6. Immune Infiltration Characterization in GSE37991

**Figure 6:**
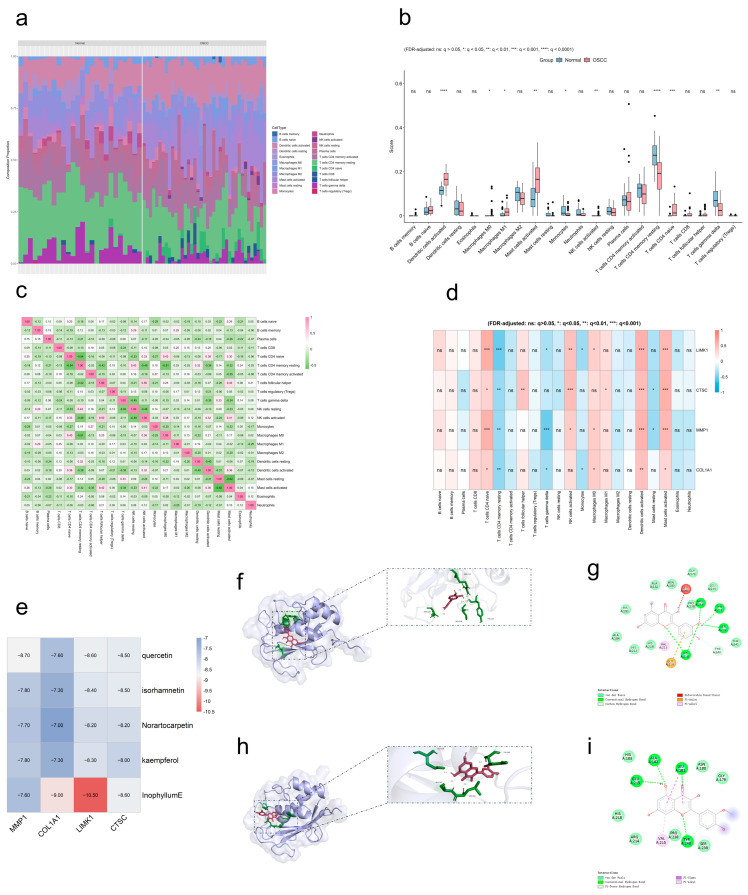
Immune infiltration analysis and molecular docking validation (OSCC). (**a**) Stacked bar graph showing immune cell infiltration in samples (GSE37991 dataset). (**b**) Box plot illustrating differential immune cell infiltration in OSCC versus normal samples. (**c**) Heatmap displaying correlations among immune cell types. (**d**) Hub gene-immune cell infiltration correlation (heatmap). (**e**) Heatmap depicting binding energies between the active ingredient and target hub protein (kcal/mol). (**f**,**g**) Quercetin-*MMP1* docking result (binding energy: −8.70 kcal/mol). (**h**,**i**) Isorhamnetin-*MMP1* docking result (binding energy: −7.80 kcal/mol).

Using CIBERSORT, we profiled immune cell infiltration in the GSE37991 dataset, quantifying the proportions of 22 immune cell types in OSCC versus healthy controls (Figure 6a). Analysis revealed strongly higher proportions of activated dendritic cells, M1 macrophages, activated mast cells, activated NK cells, naive CD4 T cells, and M0 macrophages in the OSCC group relative to controls (Figure 6b). Meanwhile, correlation analysis among immune cells revealed (Figure 6c) positive associations including resting memory CD4 T cells versus resting NK cells (r = 0.43), follicular helper T cells versus activated NK cells (r = 0.50), and naive CD4 T cells versus M0 macrophages (r = 0.43). Further analysis revealed significant correlations between the expression levels of the four hub genes and multiple immune cell infiltrations (Figure 6d), with naive CD4 T cells, resting memory CD4 T cells, activated dendritic cells, and activated mast cells showing particularly strong associations with the hub genes.

### 2.7. OSCC Molecular Docking

Molecular docking was performed to investigate the interactions between EMP active components and key hub proteins. First, we obtained four hub protein structures as receptors from PDB. Meanwhile, five crucial components (quercetin, kaempferol, isorhamnetin, norartocarpetin, and inophyllum E) screened based on network analysis were selected as ligands. The docking analysis revealed that all five crucial components exhibited significant binding ability to the key hub genes (Figure 6e). Finally, some representative docking conformations were selected for visualization (Figure 6f–i).

### 2.8. ESCC Therapeutic Targets

**Figure 7:**
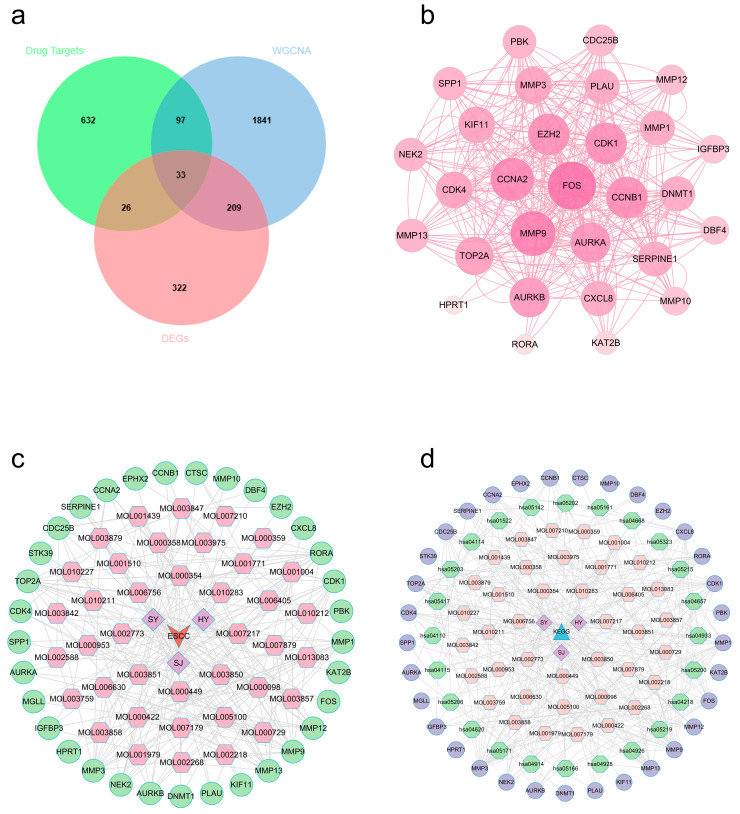
PPI and network visualization (ESCC). (**a**) Target predicted Venn diagram. (**b**) Raw PPI networks for targets. (**c**) Drug–disease network. Arrow represents esophageal squamous carcinoma, quadrilateral represents three EMPs (HY: lotus leaf, SY: mulberry leaf, SJ: sea buckthorn), hexagon represents active ingredient, and circle represents common target. (**d**) Ingredient-target-pathway network. Triangles represent KEGG, quadrilaterals represent EMPs (HY: lotus leaf, SY: mulberry leaf, SJ: sea buckthorn), hexagons represent active ingredients, octagons represent signaling pathways, and circles represent common targets.

After performing batch effect correction on the GSE44021 dataset, we found 590 DEGs through a differential expression study, then displayed them by volcano and heat figures (Figure S1a,b). Subsequently, we employed WGCNA to identify crucial modules linked to ESCC. We found that the best soft threshold for our analysis was 7, based on scale independence and average connectivity (Figure S1c,d). After creating the co-expression network, we identified modules by the dynamic tree-cutting method. In the end, we combined similar modules to obtain 12 independent modules (Figure S1e). The gene correlations within modules were demonstrated by network heatmap (Figure S1f). Module–trait association analysis showed that the turquoise module had the most significant and stable correlation with ESCC/non-ESCC phenotypes (Figure S1g). Further analysis confirmed that GS and MM exhibited a strong positive correlation for the turquoise module genes (Figure S1h). The above studies indicated that there are 2180 potential regulatory targets of ESCC. Ultimately, 33 potential therapeutic targets (Supplementary Table S3) were screened by integrating ESCC differentially expressed genes, key module genes, and active component targets of EMPs (Figure 7a).

### 2.9. PPI Network and Multidimensional Regulatory Network Construction (ESCC)

We added the identified intersecting targets to STRING platform for building one PPI graph. Cytoscape 3.10.3 was employed for visualizing it (Figure 7b). In this graph, nodes representing target proteins with higher degree values are visualized larger, indicating their more critical role in the treatment of ESCC using the three EMPs. Furthermore, we built and visualized a network that connects drugs, components, diseases, and targets (Figure 7c). Several components (e.g., quercetin, kaempferol, isorhamnetin, tetramethoxyluteolin, and inophyllum E), which have the top degree values, may play critical roles in the treatment of ESCC through multi-target synergism (Supplementary Table S5).

### 2.10. ESCC Target Functional Annotation

**Figure 8:**
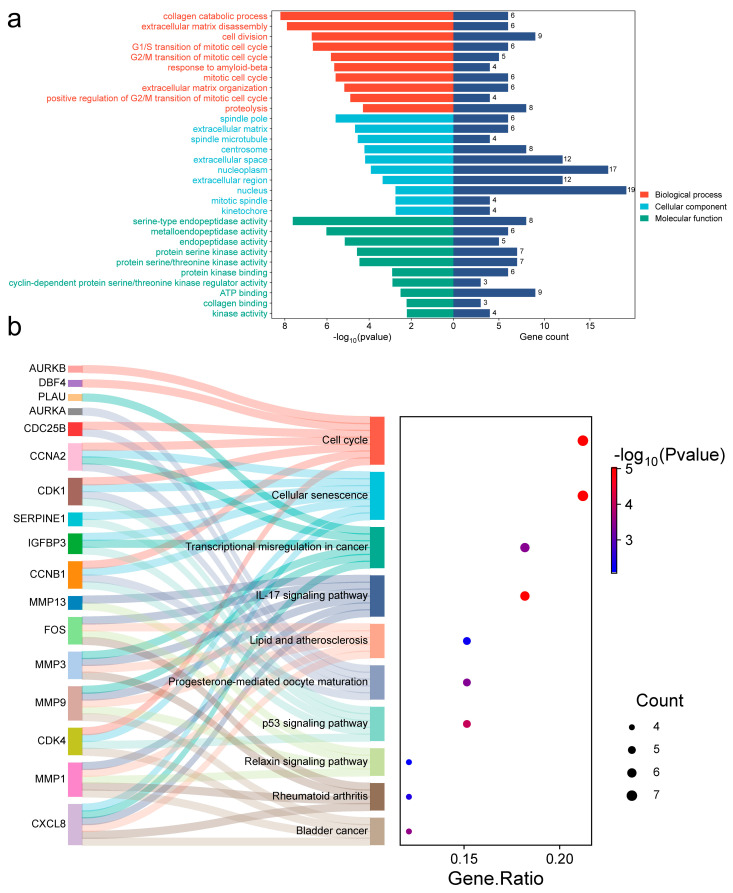
GO and KEGG analysis (ESCC). (**a**) GO profiling bilateral bar graph. (**b**) KEGG profiling bubble graph.

To investigate the underlying mechanism, we further analyzed those 33 common targets. GO analysis (Figure 8a) revealed biological processes (BP) mostly focused on the extracellular matrix disassembly, cell division, and collagen catabolic process. Cellular components (CC) were significantly enriched in spindle microtubule, spindle pole, and extracellular matrix, among others. Molecular function (MF) involved serine-type endopeptidase, metalloendopeptidase, and endopeptidase activity, among others. KEGG analysis (Figure 8b) identified significant enrichment in the cell cycle, IL-17 pathway, and cellular senescence, among others (Supplementary Table S6). A detailed mapping of candidate hub genes to their associated enriched KEGG pathways is provided in Supplementary Table S7. Using Cytoscape 3.10.3, we built constituent-target-pathway networks of the three EMPs for ESCC treatment based on these results (Figure 7d).

### 2.11. Machine Learning Screening of Pivotal Genes (ESCC)

Using the same nested cross-validation framework on GSE44021. We screened 26 core genes by SVM-RFE algorithm (*FOS*, *RORA*, *MGLL*, *CXCL8*, *DNMT1*, *CDC25B*, *SERPINE1*, *EZH2*, *SPP1*, *CDK1*, *CCNA2*, *HPRT1*, *MMP12*, *CTSC*, *CDK4*, *CCNB1*, *KIF11*, *PBK*, *KAT2B*, *PLAU*, *MMP1*, *MMP9*, *TOP2A*, *AURKA*, *MMP10*, *DBF4*) (Figure S2a,b). Additionally, the RF algorithm revealed the top nine genes ranked by significant scores (*KAT2B*, *PLAU*, *DNMT1*, *SPP1*, *MMP1*, *MGLL*, *STK39*, *HPRT1*, *TOP2A*) (Figure S2c,d). LASSO analysis determined 10 core target genes (*CDC25B*, *DNMT1*, *FOS*, *MGLL*, *MMP1*, *RORA*, *SERPINE1*, *SPP1*, *CDK4*, *IGFBP3*) (Figure S2e,f). The intersection results of the three methods showed that *MGLL*, *DNMT1*, *SPP1*, and *MMP1* may play core roles in ESCC treatment (Figure S2g). Gene correlation and expression analysis demonstrated (Figure S2h–l) that these genes are strongly correlated and exhibit significantly different expression levels in diseased versus normal groups. SHAP analysis supported feature importance (Figure S5).

### 2.12. Immune Infiltration Characterization in GSE44021

Using CIBERSORT, we profiled immune cell infiltration in the GSE44021 dataset, quantifying the proportions of 22 immune cell types in ESCC versus healthy controls (Figure S3a). Analysis revealed strongly higher proportions of M0 macrophages and M1 macrophages in the ESCC group relative to controls (Figure S3b). Meanwhile, analysis of immune cell correlations (Figure S3c) demonstrated positive relationships between: M1 macrophages and activated CD4 memory T cells (r = 0.32), resting mast cells and M2 macrophages (r = 0.32), and M1 macrophages and activated NK cells (r = 0.29). Further analysis revealed significant correlations between the expression levels of the four hub genes and multiple immune cell infiltration levels (Figure S3d), with memory B cells, M0 macrophages, M1 macrophages, monocytes, and resting mast cells showing particularly strong associations with the hub genes.

### 2.13. ESCC Molecular Docking

Molecular docking was performed to investigate the interactions between EMP active components and key hub proteins. First, we obtained six hub protein structures as receptors from PDB. Then, five core active ingredients (quercetin, kaempferol, isorhamnetin, tetramethoxyluteolin, and inophyllum E) screened based on network analysis were selected as ligands. The docking analysis revealed that all five crucial components exhibited significant binding ability to the key hub genes (Figure S3e). Finally, some representative docking conformations were selected for visualization (Figure S3f–i).

### 2.14. Cross-Cancer Analysis

**Figure 9:**
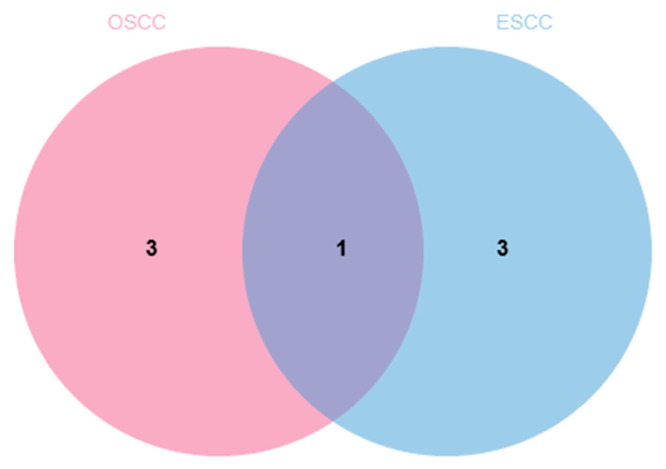
Cross-cancer analysis. Venn diagram of OSCC and ESCC.

To identify potential common hub genes in the three EMPs against squamous carcinoma (OSCC and ESCC), we conducted an intersection analysis between hub genes in OSCC (*COL1A1*, *MMP1*, *CTSC*, *LIMK1*) and those in ESCC (*MGLL*, *DNMT1*, *SPP1*, and *MMP1*). The results showed that MMP1 was the only overlapping gene, suggesting that it might be a key hub gene in the three EMPs against OSCC and ESCC (Figure 9).

### 2.15. Expression and Safety Assessment of Hub Targets

To evaluate potential targeted toxicity risks, we examined the expression profiles of key hub genes identified in this study—*COL1A1*, *MMP1*, *CTSC*, *LIMK1*, *MGLL*, *DNMT1*, and *SPP1*—across normal human tissues using the Human Protein Atlas database. The analysis revealed that *MMP1*, *COL1A1*, and *SPP1* show relatively high basal expression in connective tissues and mucosal layers, consistent with their biological roles in extracellular matrix remodeling. The remaining hub genes displayed broad yet heterogeneous expression patterns. These findings suggest that therapeutic strategies targeting these genes, particularly *MMP1*, may require tissue selectivity to minimize potential off-target effects on normal tissue homeostasis. Compared to potent single-target inhibitors, the active components from edible and medicinal plants examined in this study generally exhibit multi-target and moderate regulatory properties, which may help alleviate safety concerns to some extent.

### 2.16. Molecular Dynamics Simulation

**Figure 10:**
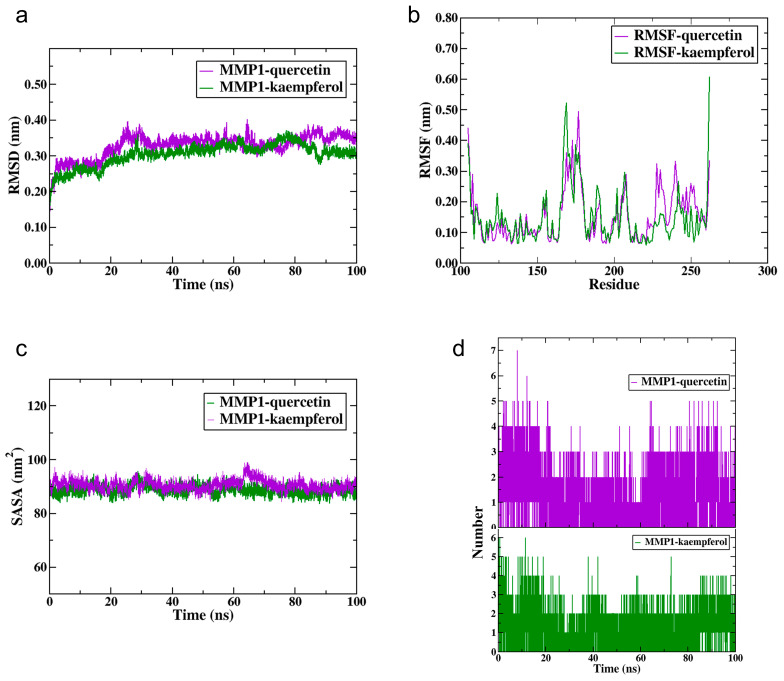
Molecular dynamics simulation. (**a**) RMSD curve. (**b**) RMSF curve. (**c**) SASA analysis. (**d**) Hydrogen bond analysis.

To conduct an in-depth investigation into the stability of protein-ligand complexes, we performed molecular dynamics simulations on the complexes formed by quercetin and kaempferol (the common components of three EMPs) with *MMP1*. The simulation results showed that both complexes maintained stable root-mean-square deviation (RMSD) values between 0.2 and 0.4 nm, indicating no significant structural changes (Figure 10a). Root-mean-square fluctuation (RMSF) analysis revealed that kaempferol binding notably increased flexibility around residue 250 of *MMP1*, whereas quercetin induced a more uniform and overall lower flexibility (Figure 10b). The solvent accessible surface area (SASA) values fluctuated moderately but remained largely within 80–100 nm^2^, suggesting consistent solvent exposure (Figure 10c). In terms of hydrogen bonds, quercetin initially formed more than kaempferol; however, both compounds stabilized at 2–4 bonds in the mid-to-late stages. Collectively, these findings demonstrate that both complexes maintained structural stability throughout the simulation (Figure 10d).

## 3. Discussion

We combined network pharmacology with bioinformatics to systematically investigate the pharmacodynamic material basis and underlying mechanisms of three EMPs—mulberry leaf, lotus leaf, and sea buckthorn—against OSCC and ESCC. Utilizing databases including TCMSP and Gene Expression Omnibus database (GEO), we identified multiple key active constituents from these EMPs, such as quercetin, isorhamnetin, and kaempferol. Accumulating evidence demonstrates that quercetin effectively inhibits cancer cell growth and invasion. It modulates key pathways, notably the IL-6/STAT3 axis, to induce apoptosis [16,17,18]. Isorhamnetin induces cell cycle arrest and apoptosis by activating the AMPK/mTOR/p70S6K pathway [19]. Through targeting the PI3K/AKT pathway and telomerase activity, kaempferol promotes apoptosis. Concurrently, its inhibition of EGFR-associated Src, AKT, and ERK1/2 signaling blocks cancer cell migration [20,21]. Although these compounds are commonly found in many plants, their consistent identification as top-degree nodes in our network analysis across three distinct EMPs suggests they may represent important shared bioactive principles contributing to the predicted synergistic effects against SCCs.

Utilizing machine learning approaches, we systematically screened and identified key hub genes specific to OSCC and ESCC. In OSCC, *COL1A1*, *MMP1*, *CTSC*, and *LIMK1* were identified as critical hub genes. In ESCC, *MGLL*, *DNMT1*, *SPP1*, and *MMP1* were identified as hub target genes. Notably, *MMP1* emerged as a shared core hub gene in both malignancies, and its expression was significantly upregulated in both OSCC and ESCC tissues, highlighting its pivotal role in squamous cell carcinoma pathogenesis. Based on the results of KEGG analysis, we found that the oncogenic mechanism for *MMP1*, a core protease responsible for extracellular matrix (ECM) degradation, exhibits tissue specificity: In OSCC, *MMP1* primarily enhances tumor migration, invasion, and metastasis via activation of the ERK1/2 signaling pathway within “Pathways in cancer” [22]. In contrast, in ESCC, *MMP1* promotes proliferation, migration, and metastasis largely via PI3K/AKT pathway activation [23]. Based on the known antitumor activities of the key EMP flavonoids and their docking affinity to *MMP1*, we hypothesize that EMP constituents may exert inhibitory effects by targeting *MMP1*. While our study did not model upstream transcriptional regulation, the literature supports that flavonoids like quercetin can downregulate *MMP1* expression via suppressing NF-κB or AP-1 signaling [17], providing a plausible indirect regulatory mechanism.

Regarding other hub genes in OSCC, research demonstrates that *LIMK1*, whose expression is suppressed by direct targeting of miR-106a, promotes cancer cell proliferation and facilitates the epithelial–mesenchymal transition process [24]. Additionally, studies have confirmed that miR-133a-3p directly targets *COL1A1* in OSCC. Suppressing *COL1A1* expression markedly inhibits the proliferation and migration of cancer cells [25]. For ESCC target genes, current evidence indicates that *DNMT1* promotes the proliferation as well as metastasis of ESCC through inactivating tumor suppressor genes RASSF1A and DAPK via hypermethylation [26]. Regarding *SPP1*, studies indicate that it contributes to ESCC progression by modulating the immunosuppressive microenvironment via GALECTIN signaling-mediated interactions with regulatory T cells [27]. Currently, experimental data elucidating the specific biological function of *CTSC* in OSCC and *MGLL* in ESCC are lacking, and their potential value as a hub gene merits further investigation.

Immuno-infiltration analysis revealed that while both OSCC and ESCC featured high infiltration of M0 and M1 macrophages, they exhibited markedly distinct tumor immune microenvironment landscapes. OSCC was characterized by enrichment of activated innate immune cells (such as dendritic cells, natural killer cells, and mast cells) and naive CD4+ T cells. The results indicate that despite both being head and neck squamous cell carcinomas, OSCC and ESCC differ significantly in the composition and regulatory mechanisms of their local immune responses, highlighting organ-specific immunomodulatory mechanisms. Furthermore, correlative profiling among immune cells identified the strongest positive association in OSCC between follicular helper T cells versus activated NK cells. This indicates that changes in the abundance of these two immune cell subpopulations within a tumor microenvironment are closely correlated, suggesting they may share a functional linkage or be subject to common regulatory mechanisms during OSCC progression. These findings warrant further investigation to elucidate their specific mechanisms of action. Furthermore, key hub genes demonstrated the most significant association with M0/M1 macrophages in ESCC. Although a direct mechanistic link between *MMP1* and immune cell recruitment was not established here, the literature suggests that *MMP1* can cleave chemokines and influence the tumor microenvironment [28]. This provides a plausible connection for future study. Molecular docking results confirmed that key bioactive natural compounds (e.g., quercetin, kaempferol, inophyllum E, and isorhamnetin) display notable binding affinity towards multiple hub proteins screened in the study. Molecular dynamics simulations further indicated stable binding between quercetin/kaempferol and *MMP1*. This finding suggests these compounds are likely key bioactive constituents contributing to the anti-squamous cell carcinoma effects exerted by the three EMPs.

Limitations: First, although the predictions of active components, targets, and mechanisms of action were based on robust bioinformatics analyses and supported by molecular simulations, they still await experimental confirmation through in vitro and in vivo studies. Second, the construction and validation of the machine learning models relied on specific GEO datasets; therefore, the generalizability of the findings needs to be strengthened through external validation using independent clinical cohorts such as TCGA data.

Future research should prioritize experimental validation of the identified core targets and key compounds in relevant OSCC and ESCC cell lines and animal models. Furthermore, exploring upstream regulatory networks (e.g., transcription factors, non-coding RNAs) that control the expression of these core genes could further deepen the mechanistic understanding. Ultimately, translating these findings toward clinical applications represents a critical direction for subsequent research.

## 4. Materials and Methods

### 4.1. Identification of Bioactive Compounds with Their Corresponding Targets

We used the TCMSP to retrieve bioactive ingredients of mulberry leaf, lotus leaf, and sea buckthorn [29], while a preliminary screening was performed according to pharmacokinetic parameters of oral bioavailability ≥ 30% and drug-likeness ≥ 0.18 [30]. We then identified effective components with corresponding target proteins. Subsequently, the obtained target proteins were searched in this UniProt platform to identify their corresponding target genes [31]. Additionally, the SMILES information of active ingredients was obtained using the Organic Small Molecule Bioactivity Database (PubChem) [32]. For components whose SMILES were unavailable in PubChem, their 3D structures were converted into SMILES using an online utility tool (https://www.novopro.cn/tools/mol2smiles.html (accessed on 20 June 2025)). Then, potential component targets were screened via Swiss Target Prediction database [33]. After integrating and deduplicating all target information above, we derived drug targets for the three EMPs.

### 4.2. Obtaining Disease Datasets

We queried the GEO with two terms, “OSCC” and “ESCC” [34]. Two datasets, GSE37991 and GSE44021, were obtained. The GSE37991 dataset contains data from 40 OSCC samples and 40 healthy samples, while the GSE44021 dataset contains data from 113 ESCC samples and 113 non-tumor samples.

### 4.3. Differential Expression Analysis

We first preprocessed the disease data using R 4.4.3. Batch effects were corrected using the sva package’s (version 3.54.0) ComBat method. We applied the limma package (version 3.62.2) [35] to screen for disease-associated DEGs, using |log2FC| ≥ 1 and an adjusted *p*-value < 0.05 as criteria (Benjamini–Hochberg FDR correction) [36].

### 4.4. WGCNA and Therapeutic Target Screening

We employed WGCNA to perform co-expression network analysis on the disease dataset [37]. We screened the top 8000 genes that have the greatest median-based dispersion to build a co-expression network. We generated Topological Overlap Matrix with the optimal soft-thresholding power and identified co-expression modules through hierarchical clustering combined with dynamic tree-cutting. Disease-associated modules were determined by module–trait relationship analysis (screening criteria: r > 0.60 with *p* < 0.05). Venn diagrams via an online platform (https://www.bioinformatics.com.cn/) overlapped differentially expressed genes, disease-module genes, and drug targets, yielding the potential therapeutic targets of EMPs.

### 4.5. PPI Network and Multidimensional Regulatory Network Construction

Those screened genes were added to the STRING database [38] to generate one PPI graph, with the species option restricted to human and other choices kept at default. After removing the disconnected nodes to filter out the core interactions, Cytoscape 3.10.3 [39] was employed for visualizing both a PPI graph and a multidimensional regulatory network integrating drug-component-disease-target relationships.

### 4.6. Functional Annotation and Component-Target-Signaling Pathway Network Construction

Intersecting targets were analyzed in the DAVID platform, employing OFFICIAL_GENE_SYMBOL for identification, with Homo sapiens specified as the species. GO functional enrichment and KEGG pathway analysis were performed. Results were illustrated by a web-based tool (https://www.bioinformatics.com.cn/) [40]. Additionally, Cytoscape 3.10.3 [41,42,43,44] was employed for drawing the multidimensional interaction graph integrating “active ingredient-core target-key pathway”.

### 4.7. Machine Learning-Driven Pivotal Gene Screening

We used an integrated machine learning strategy for feature selection of squamous cancer (OSCC/ESCC) related targets. First, SVM-RFE, RF, and LASSO regression models were built based on the R language “e1071” package (version 1.7.16), “random forest” package (version 4.7.1.2) [45,46], and “glmnet” package (version 4.1.8) [47], respectively. A nested cross-validation approach was applied to screen for genes that differentially characterize patients and healthy populations. Model performance was evaluated across outer cross-validation folds by means of receiver operating characteristic (ROC) curves and precision-recall (PR) curves, while class weighting was implemented to address sample imbalance. Subsequently, SHAP analysis was utilized to interpret the predictions of the final model. The results from the three algorithms were then cross-validated to determine a core hub gene set. Finally, gene correlation studies as well as differential expression studies were further performed.

### 4.8. Immune Infiltration Analysis

To understand how target genes affect the cancer immune environment, we estimated infiltration among twenty-two immune cells from GSE37991 along with GSE44021 datasets using the CIBERSORT algorithm [48], with 1000 permutations and retaining only samples with *p*-value < 0.05 for subsequent analyses (Input data were quantile-normalized). We used Spearman’s correlation analysis to study how different immune cell types are connected [49]. We also looked at how the levels of hub genes relate to immune cell presence. All resulting *p*-values from the correlation tests were adjusted for multiple comparisons using the Benjamini–Hochberg procedure.

### 4.9. Molecular Docking

We molecularly docked the screened active ingredients with the targets by first obtaining the structures of hub proteins and active compound ligands from the PDB [50] and the PubChem database [51], respectively. We then refined these structures with Chem3D 23.1.1.3 and PyMOL 3.1.3.1 [52,53]. Subsequently, we validated the molecular docking employing Auto Dock Vina 1.5.7 and constructed binding free energy heat maps [54]. Finally, the ligand–receptor interactions were visualized using Discovery Studio 2025 and PyMOL 3.1.3.1 [55].

### 4.10. Cross-Cancer Analysis

After obtaining machine learning screening results for OSCC and ESCC, we identified potential regulatory hub genes for these three EMPs in squamous cell carcinoma (OSCC/ESCC) through intersection analysis.

### 4.11. Molecular Dynamics Simulation

Molecular dynamics simulations were performed using Gromacs 2020.6. Topologies for the ligand and protein were prepared with the AMBER and AMBER99SB-ILDN force fields, respectively. The protein was centered in a cubic box with a minimum distance of 1.2 nm to the box edges, and NaCl ions were added to neutralize the system. Energy minimization was carried out with 50,000 steps of steepest descent. This was followed by 100 ps equilibration in both NVT and NPT ensembles. Finally, a 100 ns production MD simulation was run under periodic boundary conditions at 300 K and 1.0 bar.

## 5. Conclusions

In our work, we systematically explored the molecular mechanisms by which three EMPs (lotus leaf, mulberry leaf, and sea buckthorn) act against squamous carcinoma through active ingredients (e.g., quercetin, isorhamnetin, kaempferol, inophyllum E). Functional enrichment analysis suggested that these components could inhibit OSCC and ESCC by regulating collagen catabolism and other processes. Machine learning screening identified OSCC hub genes (*COL1A1*, *MMP1*, *CTSC*, *LIMK1*) and ESCC hub genes (*MGLL*, *DNMT1*, *SPP1*, *MMP1*), among which *MMP1* was the common hub gene in both cancers, providing a new direction for potential drug development. Immuno-infiltration analysis suggested that the active components of these EMPs could remodel the tumor microenvironment through modulation of key gene expression. Molecular simulations indicated stable binding of the primary active compound to the target. This study lays the groundwork for developing natural drugs against these squamous cell carcinomas.

## Data Availability

The original contributions presented in this study are included in the article/Supplementary Materials. Further inquiries can be directed to the corresponding author.

## Supplementary Materials

The following supporting information can be downloaded at: https://www.mdpi.com/article/10.3390/ijms27052141/s1.
