# Supplementary material for: Investigating the Mechanism of Edible Medicinal Plants Against Squamous Cell Carcinomas Based on Network Pharmacology, Bioinformatics, and Molecular Dynamics Simulation
Source: Int J Mol Sci. 2026 Feb 25;27(5):2141. doi: 10.3390/ijms27052141 (PMC12984224; doi:10.3390/ijms27052141)
Supplement: Supplementary file 1 [file ijms-27-02141-s001.zip › Supplementary Figures.pdf]

# Supplementary Materials

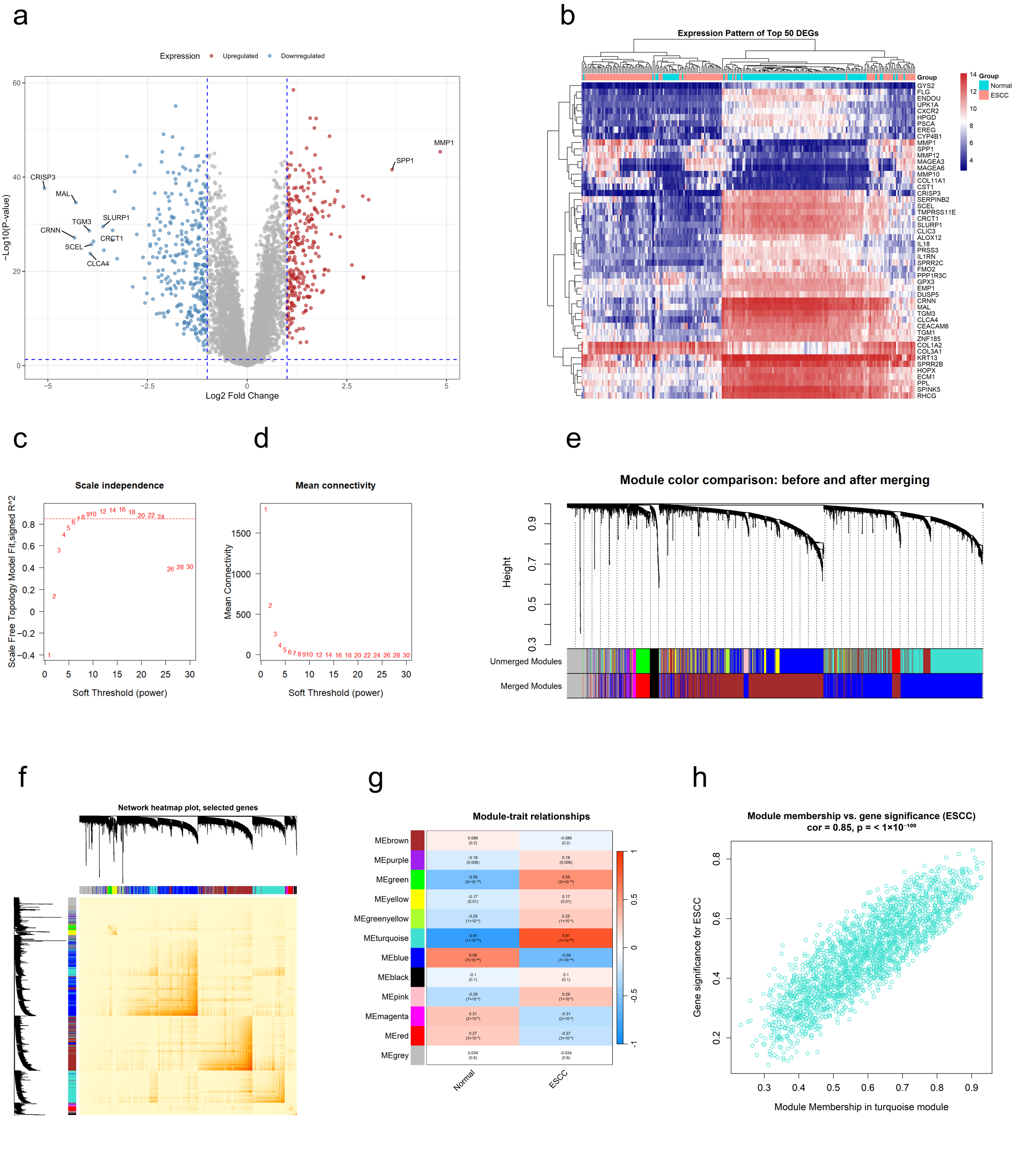


**Figure S1.** Acquisition of ESCC targets. (**a**) Volcanic map showing DEGs. (**b**) Top 50 DEGs heatmap. (**c**,**d**) Scale independence and average connectivity analysis in WGCNA. (**e**) WGCNA modules identified by color. (**f**) Gene module correlation network heatmap. (**g**) Module-trait correlations for all 12 modules. (**h**) Scatter plot of GS against MM for the turquoise module.

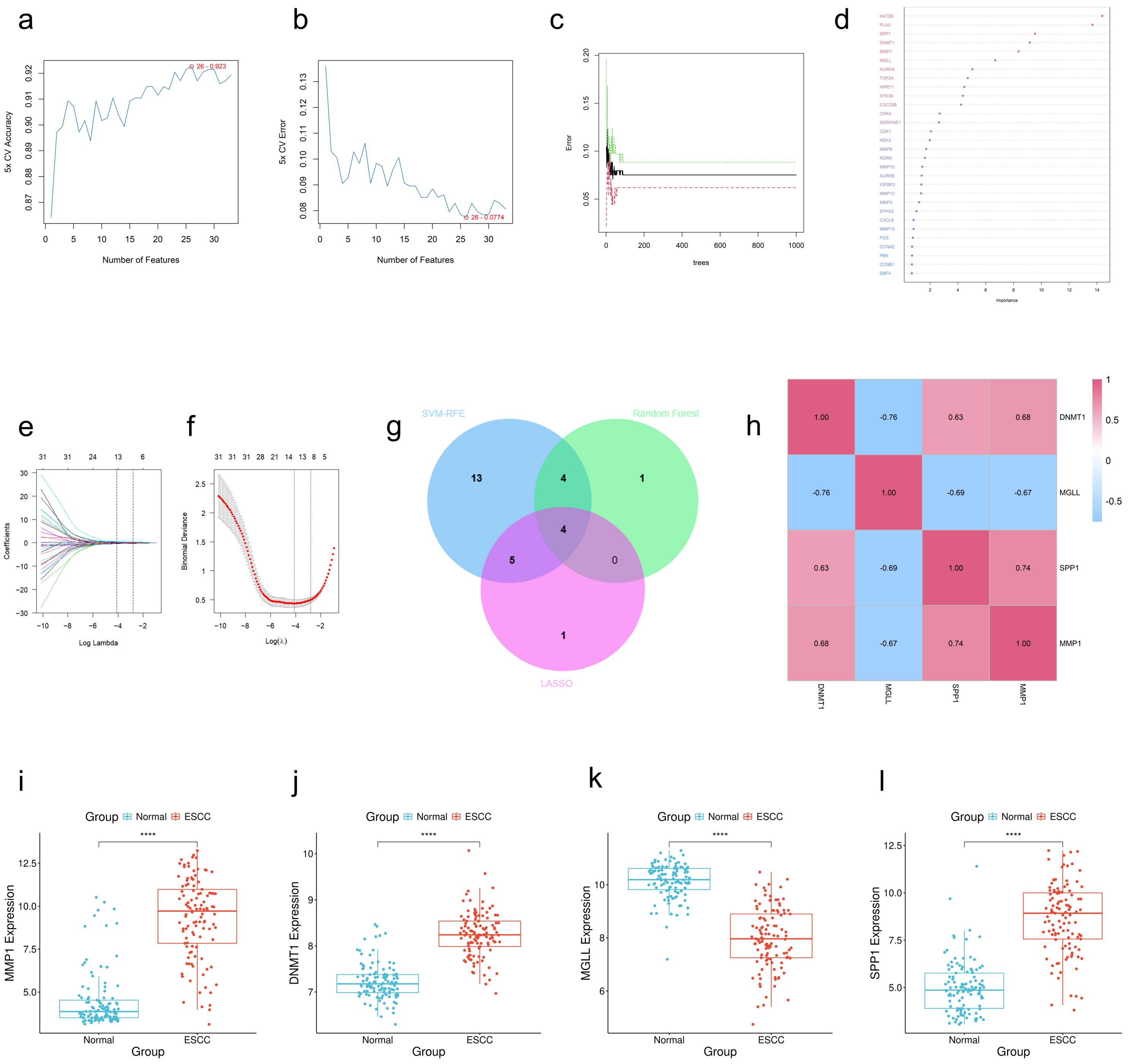


**Figure S2.** Machine learning-based hub gene screening. (**a, b**) 5-fold cross-validation accuracy vs. error rate curves for the SVM-RFE algorithm. (**c, d**) Error rate curves vs. significance assessment for the Random Forest algorithm. (**e, f**) Coefficients vs. regularized path plots for LASSO regression. (**g**) Venn diagram for key hub gene screening. (**h**) Heatmap analysis of expression correlations among hub genes. (**i-l**) Boxplots of hub gene expression levels based on the GSE44021 dataset (****: $p < 0.0001$).

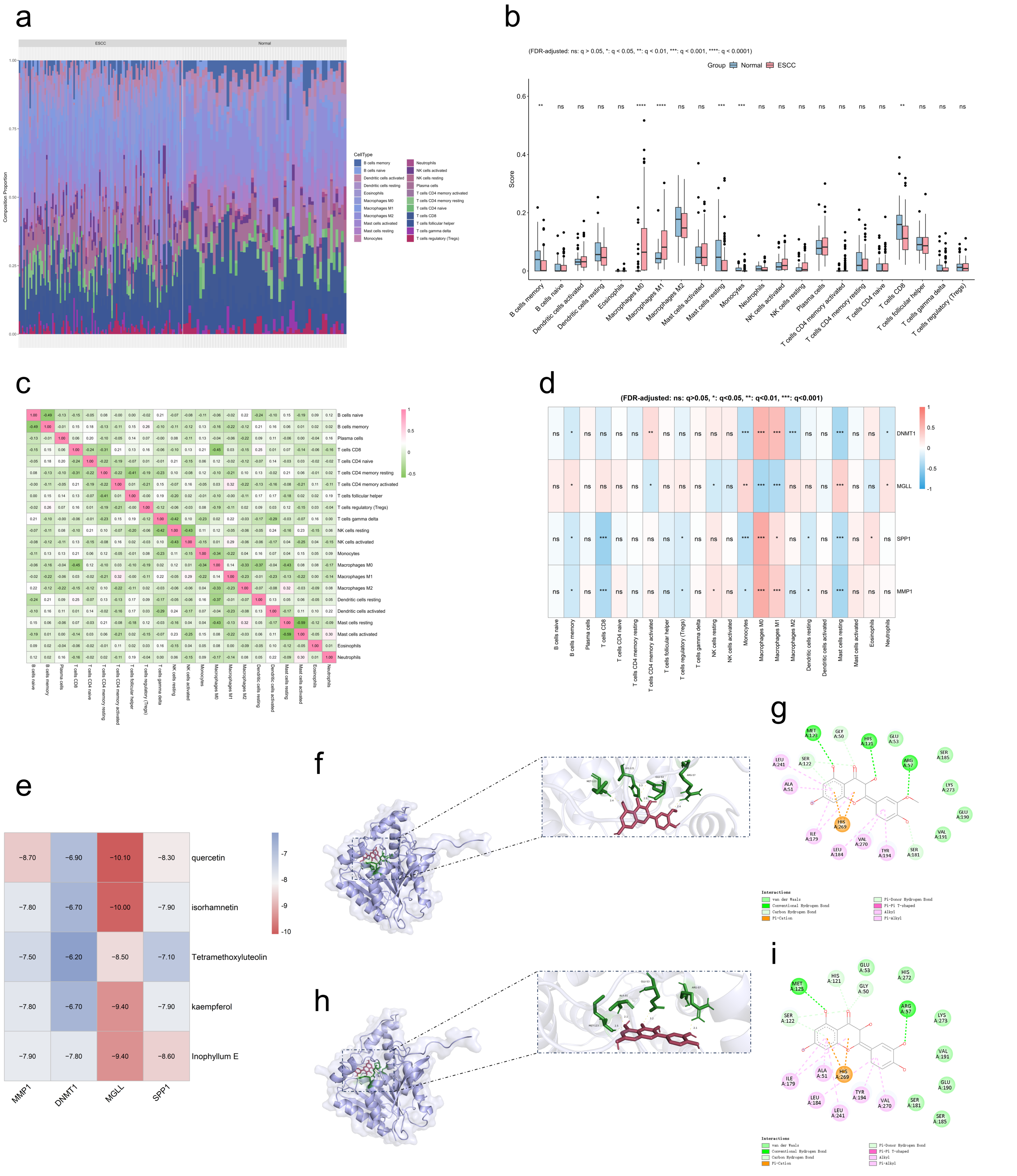


**Figure S3.** Immune infiltration analysis and molecular docking validation (ESCC). (**a**) Stacked bar graph showing immune cell infiltration in samples (GSE44021 dataset). (**b**) Box plot illustrating differential immune cell infiltration in esophageal squamous carcinoma versus normal samples. (**c**) Heatmap displaying correlations among immune cell types. (**d**) Hub gene-immune cell infiltration correlation (heatmap). (**e**) Binding energy heatmap (kcal/mol). (**f, g**) Docking pose diagrams of isorhamnetin to *MGLL* (-10.00 kcal/mol). (**h, i**) Docking pose diagrams of quercetin to *MGLL* (-10.10 kcal/mol).

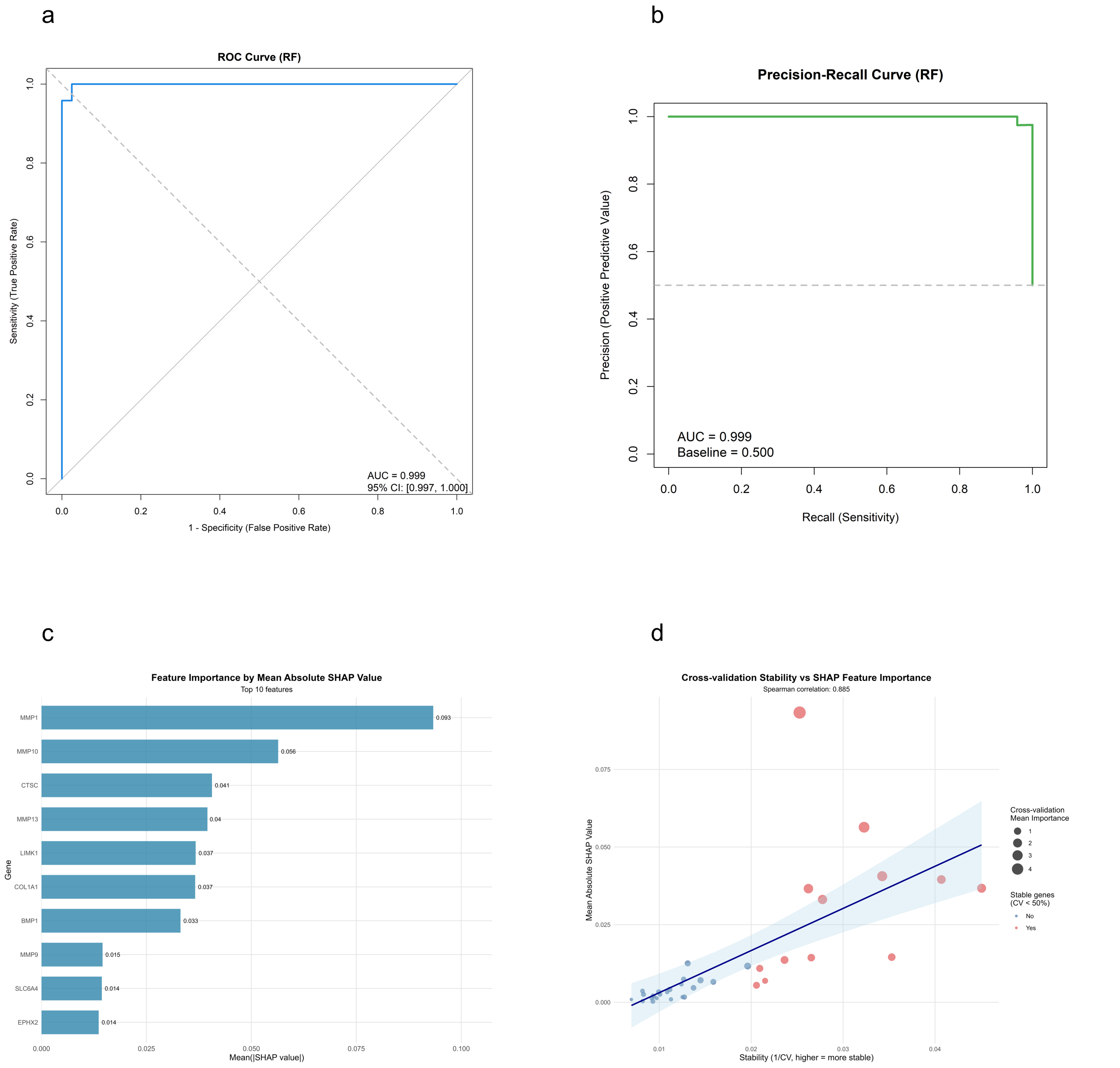


**Figure S4.** Performance evaluation of the RF model and SHAP-based feature interpretability analysis. (**a**) ROC curve. (**b**) PR curve. (**c**) feature importance bar plot. (**d**) correlation scatter plot of cross-validation stability versus SHAP feature importance for the RF model.

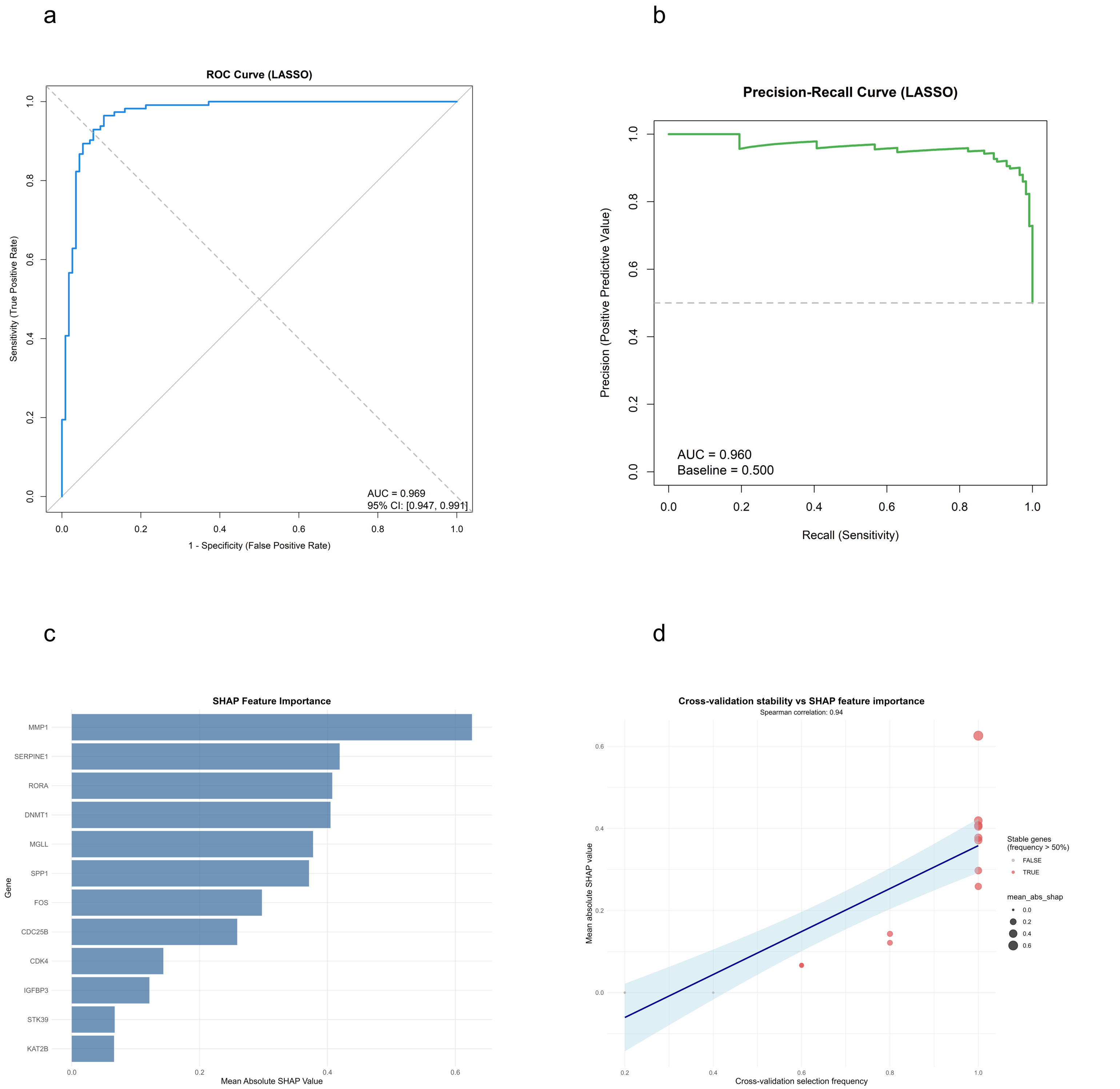


**Figure S5.** Performance evaluation of the LASSO model and SHAP-based feature interpretability analysis. (**a**) ROC curve. (**b**) PR curve. (**c**) feature importance bar plot. (**d**) correlation scatter plot of cross-validation stability versus SHAP feature importance for the LASSO model.
